# Ensuring the Safety of Chronically Ill Veterans Enrolled in Home-Based Primary Care

**DOI:** 10.5888/pcd16.180501

**Published:** 2019-09-05

**Authors:** Judith Katzburg, Debra Wilson, Jacqueline Fickel, Jason D. Lind, Diane Cowper-Ripley, Marguerite Fleming, Michael K. Ong, Alicia A Bergman, Sarah E. Bradley, Sarah A. Tubbesing

**Affiliations:** 1 VA Health Services Research and Development, VA Greater Los Angeles Healthcare System, Los Angeles, California; 2 Orlando VA Medical Center Home Based Primary Care Program, Orlando, Florida; 3 VA Health Services Research and Development Center for the Study of Healthcare Innovation, Implementation, and Policy, VA Greater Los Angeles Healthcare System, Los Angeles, California; 4 James A. Haley Veterans’ Hospital and Clinics, Research Section, Tampa, Florida; 5 VA North Florida/South Georgia Veterans Health System, Health Services Research and Development; VA Office of Rural Health, GeoSpatial Outcomes Division, Gainesville, Florida; 6 Veterans Health Administration, VA Office of Reporting, Analytics, Performance, Improvement, and Deployment, Washington, District of Columbia; 7 David Geffen School of Medicine at UCLA, Department of Medicine, Los Angeles, California; 8 Department of Health Policy and Management, UCLA Fielding School of Public Health, Los Angeles, California; 9 VA Greater Los Angeles Healthcare System, Geriatrics and Extended Care, Los Angeles, California

**Figure 1:**
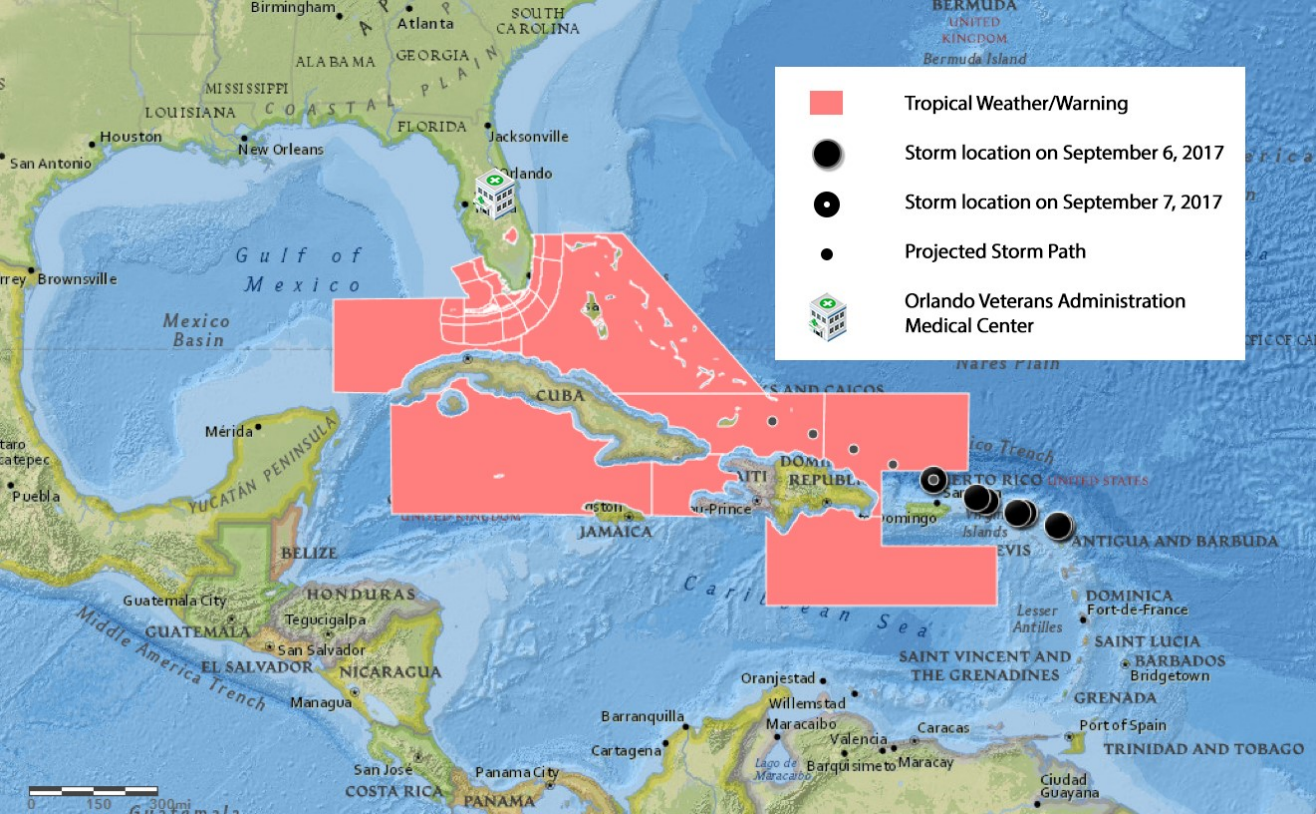
Hurricane Irma approaching Florida coastline, September 7, 2017, based on data collected September 6 and September 7, 2017. The registered nurse, a patient care manager, also served as geographic information system mapmaker (RNCM/mapmaker) for the Orlando Veterans Health Administration Home Based Primary Care program (OVAMC-HBPC), tracking the path of Hurricane Irma. Irma made landfall in the Florida Keys as a Category 4 hurricane with 132 mph winds. This powerful image of the looming threat helped inform the nurse manager, who supervised the OVAMC–HBPC nursing staff, of the severity of the storm. The RNCM/mapmaker also used the maps, in combination with patient information and other data, to educate and manage her patients. Map source: Portal for ArcGIS version 10.5 (2017), created for the Veterans Health Administration by Environmental Systems Research Institute (Esri). Additional sources: National Geographic, Environmental Systems Research Institute, Garmin, HERE Technologies, United Nations Environment World Conservation Monitoring Center, United States Geological Survey, National Aeronautics and Space Administration, European Space Agency, Micro Engineering Tech Inc., Natural Resources Canada, General Bathymetric Chart of the Oceans, National Oceanic and Atmospheric Administration, Increment P Corporation.

**Figure 2:**
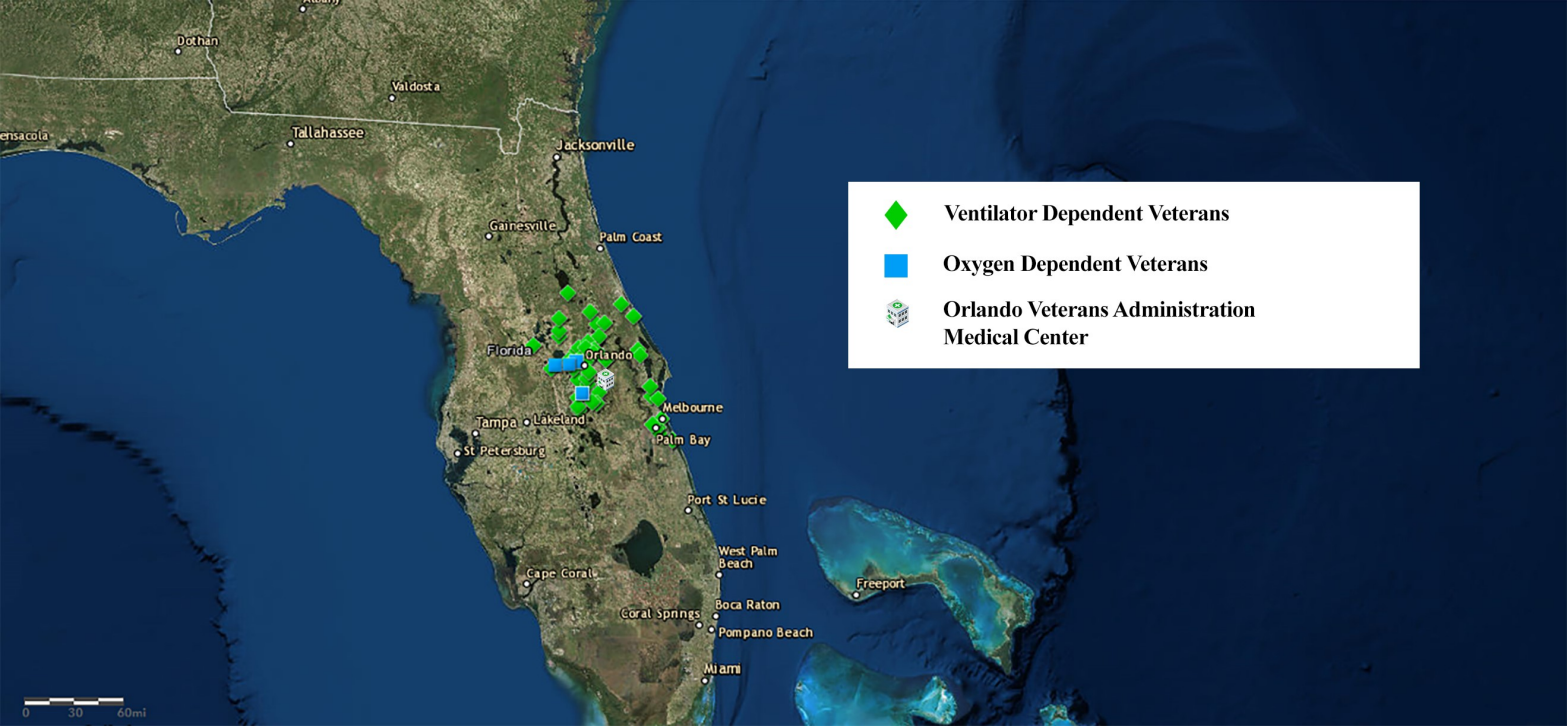
Oxygen-dependent and ventilator-dependent patients in home-based primary care, September 7, 2017. In preparation for Hurricane Irma, the nurse care manager, serving as the geographic information system mapmaker for the Orlando Veterans Health Administration Home Based Primary Care (OVAMC–HBPC) program, made maps for program leadership, including this map of oxygen-dependent and ventilator-dependent veterans. Leadership used these types of maps together with other clinical and care manager information in a dynamic process to make decisions regarding patient management in preparation for the storm. Map source: Portal for ArcGIS version 10.5 (2017) created for the Veterans Health Administration by Environmental Systems Research Institute. Additional Sources: Earthstar Geographics LLC, Environmental Systems Research Institute, HERE Technologies, Garmin.

## Background

Geographic information system (GIS) maps can be used effectively for emergency planning and response (1). Vulnerable populations, especially chronically ill older people and those dependent on medical equipment for survival, might be at particular risk during disasters (2). The use of GIS maps to plan for and respond to emergencies is becoming an important strategy for ensuring the safety of chronically ill patients (1,3,4). The Veterans Health Administration Home Based Primary Care program (VHA-HBPC) has been demonstrating the innovative use of GIS mapping for practice and patient care management through a quality improvement project, the HBPC-GIS mapping project, which is currently disseminated to 30 geographically diverse VHA-HBPC sites nationwide.

The VHA-HBPC program was designed to serve veterans with complex chronic disease (5). Home-based primary care consists of an interdisciplinary team of clinicians who provide ongoing primary care in the patient’s home (6). Veterans enrolled in VHA-HBPC are a vulnerable population, averaging more than 8 chronic conditions per patient (5). Currently, approximately 140 VHA-HBPC programs nationwide serve almost 38,000 veterans (personal communication, D. Davis, July 5, 2018).

To enhance practice management, the mapping project trains staff members at 30 VHA-HBPC programs to use VHA’s Portal for ArcGIS mapping software, version 10.5 (Esri). Self-paced, online computer-based training modules usually require several hours, with ongoing training thereafter. This novel project was designed so that any member of the VHA-HBPC staff, including frontline staff members providing direct patient care, could make maps tailored to their local program’s needs. As the mapping project expanded, evaluations indicated increasing use of GIS mapping for both emergency preparedness and response.

In 2017, some mapping project sites were adversely affected by disasters that inflicted historic costs in terms of human suffering and fiscal impact (7). For example, following Hurricane Irma, excessive heat and power outages accounted for a sizable percentage of deaths in the general population, including many elderly chronically ill patients (8). Below, we describe a case study that illustrates the innovative use of GIS maps by the Orlando Veterans Administration Medical Center HBPC Program (OVAMC-HBPC) leadership and a frontline clinical care provider to support the emergency management of patients.

## Data Sources and Map Logistics

OVAMC-HBPC joined the mapping project in 2015; a nurse care manager trained as the mapmaker (RNCM/mapmaker). Maps were created by using Portal for ArcGIS software, version 10.5 (Esri), and the RNCM/mapmaker supplied patient information. The RNCM/mapmaker incorporated several types of patient data in the map (Box), which is viewable in a popup box on the map when the cursor is moved over patient locations. Layers were added to the map indicating location of emergency services (eg, hospital). Environmental threats could be identified by additional layers (eg, hurricane path, storm surges). Event-related map layers were obtained from open sources such as the National Oceanic and Atmospheric Administration.

**Box. Patient Data Available for Incorporation into GIS Maps**

**Table:** 

Veteran’s name	**Emergency priority rating**
Last 4 digits of Social Security number	Medical foster home
Physical address	On dialysis
Zip code	On ventilator
County	Mental health provider (name)
Geocode address	Occupational or physical therapist (name)
Phone number	Dietician (name)
RN case manager (name)	Social worker (name)
Provider (Name)	Registered for special needs shelter
Patient acuity score	Lives alone
Uses oxygen	Electricity dependent

## Highlights

The map showing Hurricane Irma’s path as it approached the tip of Florida crystallized the enormity of the impending threat for the nurse manager who supervised OVAMC-HBPC nursing staff. As the storm approached, the nurse manager requested a map that identified 2 groups who might be at particular risk during a power outage: oxygen-dependent and ventilator-dependent patients. 

## Action

OVAMC–HBPC had 364 veterans enrolled in September 2017. In preparation for Hurricane Irma’s landfall in Florida, the RNCM/mapmaker frequently checked the path of the storm in Portal. The map of the oncoming storm was a powerful tool. As the nurse manager reported, “The map made me realize that it was real and it was going to come.” The RNCM/mapmaker provided requested maps to the OVAMC-HBPC program director and nurse manager, including maps showing the locations of vulnerable patients, such as oxygen-dependent and ventilator-dependent patients and patients near the coast. Maps facilitated clear and secure communication between the mapmaker and program leaders. Maps of patient locations, the storm path, and other clinical and care manager information were used by leadership in a dynamic process to make decisions regarding patient management in preparation for the storm.

As the hurricane approached, the RNCM/mapmaker used Portal to improve the quality of the care management she provided to her patients. The RNCM/mapmaker synthesized information from the GIS maps and other sources regarding the storm’s path, wind force, patient location and level of vulnerability, and areas with high likelihood of power outages. For example, with this knowledge, she effectively facilitated the sheltering-in-place of a patient with brittle diabetes by educating the patient’s daughter of the impending risk. The daughter purchased a generator to power an air conditioning unit for the patient’s room and a small refrigerator to keep his insulin cool. The RNCM/mapmaker also worked with the family of a patient diagnosed with chronic obstructive pulmonary disease and congestive heart failure who required oxygen. She convinced the family of the need for evacuation to the OVAMC hospital on the basis of the patient’s vulnerabilities identified in information from the GIS maps and from other sources.

OVAMC facilitated the transport of 23 VHA-HBPC patients to its hospital, including 2 who required admission to the intensive care unit. Because of the advanced planning of the OVAMC-HBPC and, in part, their use of GIS to integrate and analyze environmental and clinical information, fewer than 7% of their patients (23 of 364) needed to be sheltered at the hospital. No OVAMC-HBPC patient deaths or injuries were attributed to the hurricane.

In review, GIS maps in conjunction with other data informed OVAMC-HBPC leaders and facilitated care management of patients with multiple chronic diseases who possibly required emergency management before the Florida landfall of Hurricane Irma. In post-disaster assessment, the nurse manager found value in using the GIS maps and believes that maps might assist VHA employees tasked with transporting patients in future disasters (eg, by locating patients who could be evacuated together). This case study demonstrates how the use of GIS maps in emergency planning had significant benefits for patients with complex chronic conditions who receive primary medical care at home. The feasibility of having local public health departments and other home care programs provide GIS training for frontline staff in emergency management of patients is worthy of consideration.
